# Perceptions of hospitalized patients and their surrogate decision makers on dialysis initiation: a pilot study

**DOI:** 10.1186/s12882-018-0987-1

**Published:** 2018-08-08

**Authors:** Amar D. Bansal, Nina R. O’Connor, David J. Casarett

**Affiliations:** 1 Renal-Electrolyte Division, Section of Palliative Care and Medical Ethics, University of Pittsburgh School of Medicine, 200 Lothrop St, Suite C1100, Pittsburgh, PA 15213 USA; 2 Department of Medicine at the University of Pennsylvania Perelman School of Medicine, Philadelphia, PA USA; 3 Department of Medicine at Duke University School of Medicine, Durham, NC USA

**Keywords:** Acute kidney injury, Dialysis, Continuous renal replacement therapy, Decisional capacity, Decisional satisfaction, Shared decision-making

## Abstract

**Background:**

Dialysis is often initiated in the hospital during episodes of acute kidney injury and critical illness. Little is known about how patients or their surrogate decision makers feel about dialysis initiation in the inpatient setting.

**Methods:**

We conducted a prospective cohort study at a large academic center in the United States. All patients who initiated dialysis during a 30-day period in 2016 were approached for enrollment. Study participants were defined as individuals who provided consent for dialysis initiation – either the patient or a surrogate decision-maker. Decisional satisfaction and the degree of shared decision-making were assessed using the decisional attitude scale and the control preferences scale, respectively. These scales were incorporated into a study questionnaire along with an exploratory structured interview.

**Results:**

A total of 31 potential participants were eligible and 21 agreed to participate in the study. Continuous renal replacement therapy was used in 14 out of 21 cases (67%) and there was 33% in-hospital mortality in the study cohort. A majority (62%) of patients were unable to participate in the consent process for dialysis initiation and had to rely on a surrogate decision-maker. The mean score for the decisional attitude scale was 4.1 (95% CI 3.8–4.3) with a score of 5 corresponding to high decisional satisfaction. Most of the decisions were classified as shared and incorporated input from clinicians as well as patients or surrogates. Although 90% of participants agreed that they had a choice in making the decision, 81% were unable to mention any alternatives to dialysis initiation.

**Conclusions:**

Dialysis initiation was associated with high decisional satisfaction and most participants felt that the decision incorporated input from patients and providers. However, inpatient dialysis initiation was commonly associated with loss of decisional capacity and reliance on a surrogate decision-maker. This finding is likely driven by critical illness. Survivors of critical illness who remain dialysis dependent may need to revisit conversations about the rationale, risks, and benefits of dialysis.

**Electronic supplementary material:**

The online version of this article (10.1186/s12882-018-0987-1) contains supplementary material, which is available to authorized users.

## Background

Over 500,000 individuals receive dialysis for end stage renal disease (ESRD) in the United States [1]. In the absence of clinical emergencies or overt uremia, dialysis is routinely initiated in the outpatient setting. However, many patients initiate therapy during a hospitalization for acute illness. A recent study showed that among veterans this proportion may be as high as 75% [2]. Decisions to initiate dialysis in the hospital often take place in the context of acute kidney injury (AKI) with concurrent critical illness, a situation associated with high mortality.

Studies in the outpatient setting suggest that patients feel unprepared for starting dialysis and may regret the decision [3, 4]. However, the inpatient setting poses unique challenges to informed decision-making, patient autonomy, and shared decision-making. Outpatient dialysis initiation occurs exclusively among patients who have chronic kidney disease and are followed by a nephrologist. However, critical illness leading to severe AKI is restricted to the hospital setting. Therefore, the mechanisms through which patients end up on dialysis are distinct. For patients in intensive care, decisional capacity is often compromised [5] and informed consent for interventions like dialysis may be obtained from a surrogate decision-maker (SDM).

To our knowledge, there are no studies examining how patients or their SDM’s feel about dialysis initiation in the hospital setting. There is also little known about how often hospitalized patients can participate in the decision to initiate dialysis. Therefore, we conducted a prospective, questionnaire-based study of hospitalized patients with AKI requiring dialysis to assess decisional satisfaction, degree of shared decision-making, and patient participation in the decision to initiate dialysis. A short, structured interview was also utilized for exploratory purposes to gain insight into perceptions of the process of informed consent for dialysis initiation.

## Methods

### Setting and patient recruitment

The study was conducted at a large, academic referral center in the Northeastern United States (Hospital of the University of Pennsylvania, Philadelphia, PA). Patients who consented to dialysis initiation for AKI during a one-month period in 2016 were prospectively approached for enrollment into this study. Dialysis initiation was defined as the first treatment using either intermittent or continuous dialysis during a patient’s lifetime and instances were reported to study authors by consulting nephrology teams. Patients with ESRD or any previous dialysis were excluded. There were no instances of inpatient initiation of peritoneal dialysis during the study period. The participant for this study was the individual who provided consent for dialysis. Thus, in cases where the patient was unable to consent, the patient’s SDM became the study participant. To minimize the effect of outcome, hindsight, and recall bias on responses to study questions, all participants were approached within 72 h of providing written consent for dialysis. All patients had already initiated dialysis when they (or SDM’s) were approached for enrollment and they were always approached during daytime hours. Their medical record was not accessed unless they provided consent to enroll into the study.

### Design of the study questionnaire and structured interview

The study questionnaire included previously validated tools. The Decision Attitude Scale (DAS) was used to measure post-decisional satisfaction among patients or their SDM’s. This tool – along with a nearly identical one called Satisfaction With Decision scale – was developed for situations when there are difficult choices and future consequences or outcomes are unknown [6, 7]. Since the clinical outcome is unknown at the time of the decision, the DAS was designed to assess how respondents feel about the *process* of making their decision. In the clinical context, it therefore provides a way to characterize the informed consent process (Table 1).

**Table 1:** Decision attitude scale and additional study questions. Responses were a 5-item Likert Scale (Strongly agree, agree, neutral, disagree, strongly disagree)

Decision attitude scale	
I had no problem using the information	
I am comfortable with my decision	
The information was easy to understand	
I wish someone else had made the decision for me	
It was difficult to make a choice	
I am satisfied with my decision	
My decision is sound	
More information would help	
My decision is the right one for my situation^a^	
Consulting someone else would have been useful	
Additional study questions	
Signing the consent form was mainly to protect the hospital	
Signing the consent form was a waste of time	
I feel like I had a choice in making this decision	
Going through the process of consent for dialysis was important to me	

^a^ This is the original wording of the Decision Attitude Scale (DAS). When the study participant was a surrogate decision-maker (as opposed to the patient), this statement was modified to read, “My decision is the right one for the situation”

To assess the degree of shared decision-making, participants and the nephrology provider who obtained consent for dialysis were also asked to complete a modified version of the Control Preferences Scale (CPS, Table 2) [8]. The CPS has been used to assess perceptions of how treatment decisions are influenced by patients’ and physicians’ preferences. Decisions are classified based on five choices that reflect a spectrum of autonomy. Fully autonomous decisions are those made exclusively by either the patient or physician. Alternatively, there can be varying degrees of shared decision-making with input from both patient and physician. By design, nephrology providers could see the CPS during the study, but to prevent the study from influencing physician behavior (Hawthorne effect), the remaining contents of the questionnaire were not revealed to them. Lastly, open-ended questions about aspects of the informed consent process for dialysis were asked as part of a structured interview (Additional file 1: Figure S1). Verbal responses were written down by the interviewer. Audio-recording was not used to maximize participant willingness to answer questions.

**Table 2:** Modified Control Preferences Scale. The original wording of the CPS can be found in reference [8]

Option	Patient perception scale	Physician perception scale
A	I made the final decision about dialysis	The patient made the final decision about dialysis
B	I made the final decision about dialysis after seriously considering the kidney doctor’s opinion	The patient made the final decision about dialysis after seriously considering my opinion
C	The kidney doctor and I shared responsibility for deciding about initiating dialysis	I shared responsibility with the patient for making the final decision about dialysis
D	The kidney doctor made the final decision about dialysis but seriously considered my opinion	I made the final decision about dialysis after seriously considering the patient’s opinion
E	The kidney doctor made the final decision about dialysis	I made the final decision about dialysis

Notably, references made to the “consent process” in questionnaire items are referencing the consent process for dialysis initiation, not the consent process for this research study. Standard practice at the study institution is to obtain written consent for dialysis initiation from all patients. These consents are typically obtained by the nephrology fellow. Participants were reminded of this distinction as needed.

### Ethics

All participants provided written informed consent. The study protocol, consent forms, and items associated with the questionnaire were approved by the Institutional Review Board in the Office of Regulatory Affairs at the University of Pennsylvania. Prior to initiating the study, the overall aims of the project were also discussed with the nephrology department. All nephrology providers active during the study period (both faculty and fellows) agreed to participate in the study.

### Data analysis and reporting

Responses from encounters with study participants were initially handwritten onto study sheets and later entered into a de-identified database (Microsoft Excel) and analyzed using Stata. To streamline data reporting, 5-item Likert scale responses were recoded into three possible responses: agree, disagree, and uncertain. Given that the motivation of this work is descriptive and qualitative, statistical analysis is limited to the computation of means and 95% confidence intervals. These calculations were performed in Stata (StataCorp, College Station, TX).

## Results

Of the 31 patients and SDM’s who met study criteria, 21 agreed to participate. Table 3 shows baseline demographics for the enrolled patients. Enrolled patients had a mean age of 63 years, one-third were African-American, and most had both pre-existing diabetes and hypertension. Medical history for potential participants who declined to enroll is not available since their records were not accessed to preserve privacy. Most patients were in an ICU and continuous venovenous hemodialysis (CVVHD) was used more often than intermittent dialysis (IHD) as the initial modality. The mean baseline creatinine for enrolled patients was 2.3 mg/dL. For 19 of the 21 patients, this baseline was defined as the immediate prehospitalization value. Study participants were supposed to be approached within 72 h of signing the dialysis consent form and all cases met this requirement. The mean time between dialysis consent and study enrollment was 36 h.

**Table 3:** Patient demographics

Patient characteristics	Total cohort (*n* = 21)
Demographics	
Age: mean (SD), years	63 (13)
Gender: *n* (% male)	15 (71)
African-American: *n* (%)	7 (33)
Care delivery	
Surgical primary team: *n* (%)	6 (29)
Medical history	
Hypertension: *n* (%)	13 (62)
Diabetes: *n* (%)	11 (52)
Baseline creatinine: *n* (SD), mg/dL	2.3 (1.8)
Initial dialysis modality	
CVVHD: *n* (%)	14 (67)
IHD: *n* (%)	7 (33)
Mortality	
Overall In-hospital mortality: *n* (%)	7 (33)
Hospice at discharge: *n* (%)	1 (5)

*SD* standard deviation, *CVVHD* continuous venovenous hemodialysis, *SDM* surrogate decision-maker, *IHD* intermittent hemodialysis

### Patient participation in the decision to initiate dialysis

Of the 21 patients included in the study, 13 (62%) were not able to participate in the decision to initiate dialysis and relied on an SDM for consent. This lack of decisional capacity is reflective of the illness severity seen among the cohort of patients studied, most of whom were treated in an ICU setting. This finding is supported by the fact that 12 out of 13 patients who initiated CVVHD relied on an SDM.

### Decisional attitudes towards consent for dialysis initiation

The mean score from the DAS for all 21 participants was 4.1 (95% CI 3.8–4.3), with a score of 5 implying the highest post-decision satisfaction. In addition, 17 (81%) participants agreed that “going through the process of consent for dialysis was important” to them. All 21 participants disagreed with the statement that “signing the consent form was a waste of time.” Lastly, 19 (90%) participants felt like they “had a choice in making this decision.” In contrast to this sense that participants valued the consent process, 9 (43%) participants agreed that “signing the consent form was mainly to protect the hospital.”

This study was not designed based on thresholds for statistical power to detect differences in mean DAS scores through stratified analyses. However, there was no statistically significant difference in DAS scores when comparing across dialysis modality or the presence of an SDM (Table 4).

**Table 4:** Mean decisional attitude score stratified by dialysis modality (a) and presence of an SDM (b)

	Mean DAS score with 95% CI
a) Dialysis modality	
iHD (*n* = 8)	3.9 (3.4–4.4)
CVVHD (*n* = 13)	4.1 (3.8–4.5)
b) Surrogate decision maker	
Absent (*n* = 8)	3.9 (3.4–4.3)
Present (*n* = 13)	4.2 (3.8–4.6)

*DAS* decisional attitude scale, *CI* confidence interval

Participants’ responses to structured interview questions are shown in the Additional file 2: Table S1. Although a formal thematic analysis was not performed, responses suggest that there were a variety of feelings and emotions about the circumstances of dialysis initiation. 19 (90%) participants mentioned toxin removal, cleaning, purification, or volume removal when asked to summarize what they knew about dialysis. Consistent with their high DAS scores, most participants (11 out of 21, 52%) had feelings of gratitude for care received.

### Alternatives to dialysis initiation

Although 19/21 (90%) of participants felt like they “had a choice” in making the decision to initiate dialysis, 17 (81%) answered that they were unaware of any alternatives to dialysis (Additional file 2: Table S2). Two participants, (9%) alluded to waiting a longer time to see if there was renal recovery, and 2 (9%) mentioned either kidney transplant or another dialysis modality. Of the 17 patients who identified no alternatives to dialysis, two specifically mentioned death in their response to the question.

### Perceptions regarding shared decision-making

**Fig. 1:**
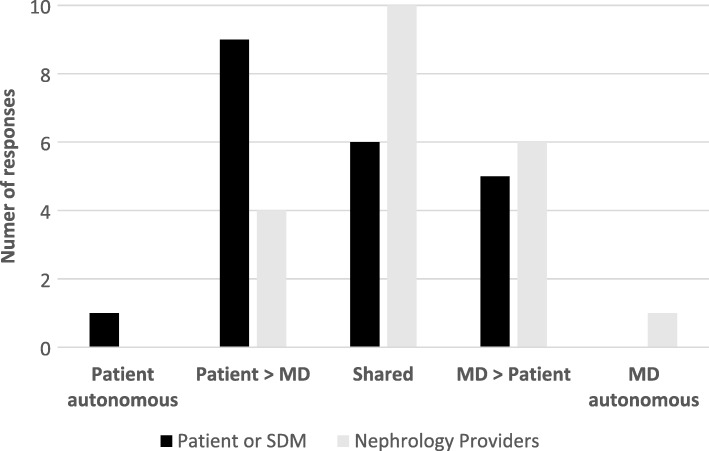
Distribution of responses to Control Preferences Scale (21 pairs). All responses to Control Preferences Scale (CPS) by 21 paired sets of study participants and corresponding nephrology providers. The categories on the horizontal axis correspond to selections on the CPS, which is shown in Table 2. Choice A from the CPS corresponds to “Patient autonomous,” while choice E corresponds with “MD autonomous”

**Fig. 2:**
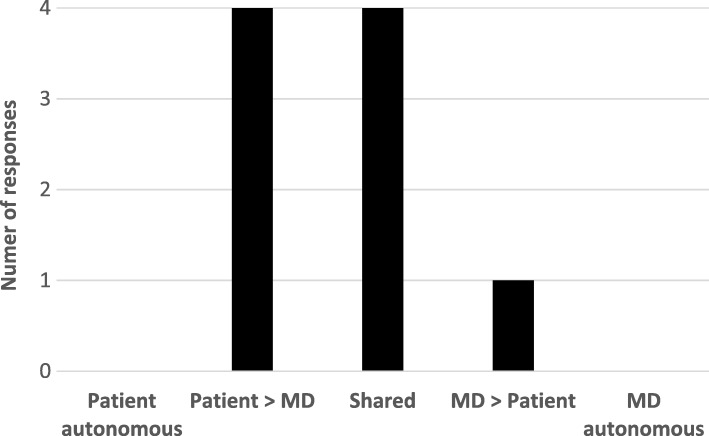
Distribution of concordant responses to Control Preferences Scale (9 pairs). Concordant responses refer to when a patient/SDM and nephrology provider chose the same response to the Control Preferences Scale. From the 21 paired sets, there were 9 concordant pairs, which are shown here

Study participants and their corresponding nephrology providers were both asked to select one of five choices from the modified CPS (Table 2). The distribution of these paired responses is shown in Fig. 1. Most of the responses indicated that dialysis initiation was either an equally shared decision, or one that incorporated some input from both the patients/SDM’s and nephrology providers. Of the 21 paired responses, there were 9 (43%) pairs in which responses were concordant (patient/SDM and provider chose the same item from five choices). Figure 2 shows the distribution of these concordant responses. Among concordant pairs, none felt dialysis initiation was an autonomous decision and most felt that the decision was shared.

## Discussion

Dialysis is often initiated in the hospital when AKI occurs in the setting of critical illness. When AKI is severe enough to require dialysis, there is a high prevalence of conditions like sepsis, the use of vasopressors, and mechanical ventilation [9]. Thus, when compared to individuals who start hemodialysis as outpatients, hospitalized patients face a different clinical scenario for decision-making. This study prospectively approached all hospitalized patients with AKI requiring dialysis at a large, tertiary medical center for 30 consecutive days. There was predominant use of a continuous dialysis modality (67%), a therapy that can only be performed in an intensive care unit (ICU). Consistent with prior reported outcomes, the study cohort had high in-hospital mortality (33%) [10, 11]. One of the main findings is that 62% of patients could not participate in the decision to initiate dialysis and relied on a SDM. This reliance on a SDM is likely a reflection of the general loss of decisional capacity commonly seen in intensive care. Although this study did not assess for patient participation in other decisions like mechanical ventilation, insertion of central venous catheters, or enteral feeding tubes, a high percentage of critically ill patients rely on SDM’s for multiple decisions, especially near the end of life [5].

The fact that more than half of the decisions to initiate dialysis were made by SDMs and not the patients themselves stresses the overall importance of being aware of patients’ values and preferences. Ideally, gaining such insight would take place prior to clinical decompensation and be part of longitudinal advance care planning.

For patients who survive their acute illness and remain dialysis dependent, our study findings suggest that most of them may need to revisit the risks, benefits, and rationale of dialysis since they were not able to participate in the initial discussion. In our experience, due to the structure of inpatient care, which may involve multiple handoffs, this type of conversation often does not take place.

Study participants were essentially unable to identify any alternatives to dialysis. Alternatives could include time-limited trials, conservative care, comfort-directed care, de-escalation of care, palliative care, or hospice. A lack of awareness of alternatives to dialysis is especially noteworthy considering the high mortality seen in the study cohort. Based on the data presented here, it is not possible to extrapolate what drives this inability to articulate alternatives to dialysis. However, we speculate that in the context of acute illness, clinical momentum favoring continuation of life-sustaining therapies likely framed the decision to initiate dialysis as “necessary” and more conservative options were probably not presented. It is also possible that participants were unable to recall a discussion of alternatives to dialysis, but we feel this is less likely.

Acute initiation of dialysis in the United States relies mainly on the hemodialysis modality. Data presented here are consistent with this trend as there were no patients who initiated on peritoneal dialysis. Prior work has shown dialysis modality selection can be influenced by the timing of nephrology referral, with late referral being less likely to be associated with the use of peritoneal dialysis [12, 13]. Therefore, our findings suggest that patients who initiate dialysis in the hospital may need heightened attention to modality education since hemodialysis is essentially used as the “default” therapy in most acute settings.

This work has some limitations. The data are from a single center in the United States and may not be representative of larger trends. Due to resource limitations, prospective enrollment into the study was pre-determined to continue for 1 month. In addition, although the study questionnaire was aimed at assessing aspects of nephrology care delivery, patients or SDM’s may not have been able to rate the nephrology team in isolation of their entire hospital or ICU experience. Therefore, although an effort was made to approach study participants within a short window of dialysis initiation, their responses to questionnaire items is likely influenced by external factors. The high score on the DAS reported in this study could be related to the fact that study participants who were very unsatisfied with care or were extremely ill declined to participate when they were approached for enrollment. It is difficult for us to comment on the prevalence of chronic illness in the study cohort because the protocol for medical record review did not include an assessment for underlying cardiovascular disease or cancer. Lastly, there may have been a Hawthorne effect prompting positive behavior change by nephrology teams resulting in higher than expected DAS scores.

## Conclusions

This study adds important insight into the largely unexplored questions surrounding decisional capacity and decisional satisfaction for dialysis initiation in hospitalized patients. Although additional studies are needed, our data suggest that a substantial percentage of patients who initiate dialysis in the hospital may be unable to participate in that decision. In addition, despite the high mortality associated with severe AKI requiring dialysis initiation, patients and their SDM’s are either not being told about alternatives to dialysis or cannot recall a discussion about alternatives. Further qualitative work is needed to clarify how patients who survive critical illness with ongoing dialysis needs feel about various aspects of their care. This kind of inquiry can help improve the delivery of patient-centered care, especially among those patients who emerge from acute illness with ESRD.

## Additional files

Additional file 1: **Figure S1.** Structured interview questions. (DOCX 14 kb)

Additional file 2: **Table S1.** Responses to structured interview questions. (PDF 371 kb)

## Abbreviations

AKI: Acute kidney injury
CPS: Control preferences scale
CVVH: Continuous venovenous hemodialysis
DAS: Decisional attitude scale
ESRD: End stage renal disease
IHD: Intermittent hemodialysis
SDM: Surrogate decision maker

## References

[1] United States Renal Data System (2017). Annual data report: atlas of chronic kidney disease and end-stage renal disease in the United States.

[2] Wong S, Vig E, Taylor J, Burrows N, Liu C-F, Williams D (2016). Timing of initiation of maintenance dialysis: a qualitative analysis of the electronic medical records of a national cohort of patients from the department of veterans affairs. JAMA Intern Med.

[3] Song M-K, Lin F-C, Gilet C, Arnold R, Bridgman J, Ward S (2013). Patient perspectives on informed decision-making surrounding dialysis initiation. Nephrol Dial Transplant.

[4] Davison S (2010). End-of-life care preferences and needs: perceptions of patients with chronic kidney disease. Clin J Am Soc Nephrol.

[5] Silveira M, Kim S, Langa K (2010). Advance directives and outcomes of surrogate decision making before death. N Engl J Med.

[6] Sainfort F, Booske BC (2000). Measuring post-decision satisfaction. Med Decis Mak.

[7] Holmes-Rovner M, Kroll J, Schmitt N, Rovner DR, Breer ML, Rothert ML (1996). Patient satisfaction with health care decisions: the satisfaction with decision scale. Med Decis Mak.

[8] Degner LF, Sloan JA, Venkatesh P (1997). The control preferences scale. Can J Nurs Res.

[9] Grubbs V, O’Riordan D, Pantilat S (2017). Characteristics and outcomes of in-hospital palliative care consultation among patients with renal disease versus other serious illnesses. Clin J Am Soc Nephrol.

[10] Ghahramani N, Shadrou S, Hollenbeak C (2008). A systematic review of continuous renal replacement therapy and intermittent haemodialysis in management of patients with acute renal failure. Nephrology.

[11] Clermont G, Acker CG, Angus DC, Sirio CA, Pinsky MR, Johnson JP (2002). Renal failure in the ICU: comparison of the impact of acute renal failure and end-stage renal disease on ICU outcomes. Kidney Int.

[12] Schmidt R, Domico J, Sorkin M, Hobbs G (1998). Early referral and its impact on emergent first dialyses, health care costs, and outcome. Am J Kidney Dis.

[13] Smart NA, Titus TT (2011). Outcomes of early versus late nephrology referral in chronic kidney disease: a systematic review. Am J Med.

