# Supplementary material for: Perceptions of hospitalized patients and their surrogate decision makers on dialysis initiation: a pilot study
Source: BMC Nephrol. 2018 Aug 8;19:197. doi: 10.1186/s12882-018-0987-1 (PMC6083629; doi:10.1186/s12882-018-0987-1)
Supplement: Supplementary file 1 — Figure S1. Structured interview questions. (DOCX 14 kb) [file 12882_2018_987_MOESM1_ESM.docx]

**Figure S1 – Structured interview questions**

For the past 3 to 4 days, what has been the most important decision ([you have had to make?] or [you have had to make for your relative/friend?]) *^1^*

Describe your feelings about dialysis and the way in which it was started.

Can you summarize what you know about dialysis?

What is your understanding about why ([you need] or [your relative/friend needs]) dialysis? *^1^*

What alternatives to dialysis ([do you] or [your relative/friend]) have? *^1^*

What feedback do you have for the nephrologists (kidney doctors) ?

1. Wording for patients and surrogate decision-makers, respectively, is shown in brackets.
