# Supplementary material for: Perceptions of hospitalized patients and their surrogate decision makers on dialysis initiation: a pilot study
Source: BMC Nephrol. 2018 Aug 8;19:197. doi: 10.1186/s12882-018-0987-1 (PMC6083629; doi:10.1186/s12882-018-0987-1)
Supplement: Supplementary file 2 — Table S1. Responses to structured interview questions. (PDF 371 kb) [file 12882_2018_987_MOESM2_ESM.pdf]

Table S1

study ID number	For the past 3 to 4 days, what has been the most important decision you have had to make (for your relative/friend)?	Describe your feelings about dialysis and the way in which it was started	Can you summarize what you know about dialysis?	What is your understanding of why you (your relative/friend) need(s) dialysis?	What alternatives to dialysis do you have?	What feedback do you have for the nephrologists (kidney doctors)?
17	How we are going to proceed about dialysis	A little bit of apathy	It's going to clean out my blood	Because my blood is becoming toxic	None	Do a good job. It took a while to discover what was wrong.
15	I had to get my leg looked at…it's important for me to know what is wrong with it	I feel complete	It's supposed to help make me better	Because they want me to live	I don't	You need more like Wasserstein. He's compassionate.
40	The DNR decision	I had no idea about how it works. It's scary to me. The discussion made me understand it better.	It's going to cleanse his body.	Because things are moving. He is not able to urinate.	We weren't given any.	I like the way a doctor sat here for 10 minutes and spoke to me. This one doctor broke it down for me.
38	Calling 911 to bring him to the hospital	Grateful.	Filters the toxins out of the blood.	Because the leukema cells break down and deposit crap and mess up the kidneys.	There are none.	I don't recall any discussion about risk.
44	Dialysis.	Happy because the toxins would be removed. And glad.	It purifies the blood.	To remove toxins - kidneys were weak.	None	Great as a team. Provide good service. Explain things well.
42	Dialysis.	Concerned.	A system used to clean the blood and rest the kidneys and to draw fluid.	He needs to have blood cleaning to reduce BUN and creatinine levels. Also, he needs to draw off fluid.	Could have waited and done nothing and see if his kidneys would have woken up.	I'm appreciative of explanations.
13	I've had no decisions decisions to make, honestly. My only kidney is coming out. I need this.	I didn't have any pain whatsoever. Perfectly fine.	It purifies the blood and puts it back into the body. Because the kidneys can't do it anymore.	Because I'll have no kidneys.	There is no alternative.	I don't know. I love being here. Everybody is so nice.
7	I don't know.	Scared.	A machine filters my blood because my kidneys cannot.	Because my kidneys are ruined.	None	Happy with my care.
11	My relationship with God.	Pleased and slightly puzzled.	Is a process of removing toxins and wastes and other substances from the body and hopefully replacing them with other cleaner substances.	Because I keep accummalating lots of negative chemicals.	None, now.	Continue to study!
9	Having the Swan [ganz catheter] put in.	Good.	Cleaning my blood. It's my only option.	My kidney function is bad.	None	They're all good.

30	Bringing him to the hospital.	Necessary.	A process to remove toxins from the blood when the kidneys are unable to do so on their own.	He was in metabolic acidosis and was acidemic with an elevated lactic acid and had ATN. Removal of toxic acids was necessary because he had ATN from sepsis.	None - death.	They were kind.
24	The idea that I had to shut down my mothers body and let machines heal her.	Very mixed. Result was wonderful, but [in] hindsight I feel more thought should have been given.	It's like a washing machine. Filtering and purifying the blod. Getting rid of the toxins.	Because her kidneys are shut down.	There were none.	The maches that are provided are antiquated. The machines were breaking down - they're not up to date. Nurses were getting very frustrated. I spoke to 2 different kidney [doctors] on the weekends. I wonder if someone has seen a kidney doctor - would they look that up? She sees Dr. Rudnick.
22	I would probably say the dialysis, but it's hard to say because I've been asked to give consent for 3 to 4 things which they haven't done yet.	I had a negative connotation about it because the other hospital seemed like they were mismanaging him. So I thought it was an extreme thing.	Machine takes your blood out, cleans it, filters it, and puts it back into your body.	Because the extreme drops in his BP let his kidneys go into shock. Being defibrillated also caused his kidneys to go into shock. And he isn't getting fluid off in a good enough rate.	None. Lasix and other meds weren't working.	I don't have enough data right now so I don't have any feedback. But nothing negative. When they did consent on the telephone it was very thorough.
5	Where I was going to start my regular dialysis after I left the hospital.	I have no issues with the way it was started. It sucks, but if I want to live I have to go to dialysis.	My kidneys aren't functioning so what dialysis does is it basically purifies the impurities in my blood. The machine takes it out, cleans it, and puts it back in.	Because my kidneys aren't functioning.	We've spoken about transplant. There's HD and PD. There's basically 2 choices. Or death.	I don't have any negative feedback. No problems with Dr. Denker either.
20	I believe that all the procedures that have been recommened [multiple abdominal surgeries] are important. The whole process altogether.	At first I thought it was going to be a typical session (3 times per week) but the doctor said we'd do it gently. [I'm] at ease where the staff are so proactive. The hospital is renowned. [I] feel fortunate.	A process where they clean the blood and remove toxins when the kidneys can't.	Because he's on medication that is affecting his BP and blood flow is limited to his vital organs. He was receiving chemo and got sepsis...had trouble breathing...low BP...	None	No input in regards to how to better/improve anything. Very respectful. Had not seen one before.

18	I've not made any important decisions. I've listened to the doctors and their advice was sound.	I think it's lifesaving.	Correct his fluid overload and hopefully maintain his electrolytes and rest his kidneys.	Het's got ATN - acute renal failure.	We've been waiting...72 hours...[he's] not recovering...diuretics...	No feedback. [I'm] very appreciative.
14	Consent for the first surgery [subdural evacuation].	I know it's necessary but I'm disappointed. We thought we'd be going directly to transplant and we're afraid he'll be on it until transplant.	Process where toxins are washed from the blood and blood is returned to the body.	He had 25 plus years of high BP medicines and other medicines that have ruined his kidney functions.	He has been on a sodium-free diet. Transplant is our goal. We have six donors lined up.	To be gentful when you explain that kidney damage doesn't get better. That was difficult.
10	I think the only one in the last 3 to 4 days was the dialysis decision.	First of all I was worried. Didn't know a lot about [it]. Unsure of implications of it.	Exchange of blood - to clear out detrimental products...almost like cleaning it. I understand it's hard on the patient.	The content of his blood was not what the medical profession wanted to see.	I don't know	Haven't been here to talk to them personally. Sometimes they talk too fast. Not particular to one person. The nurses take a lot of tim to talk to you.
3	Dialysis.	It something that had to be done.	I thought it was a good experience. I don't know a whole lot abou tit. It helps clean the liver.	To keep me healthy.	Dying.	None.
8	I don't know.	I wish he didn't have to have it. If it's necessary it has to be done.	...is that a line has to be put in and that blood has to be taken out for purification [for] the kidney in order for it to function better.	So that he can live.	At this stage there is no other alternative.	I don't know.
2	At one point they asked about "pulling [the] plug." Conitnuing ICU treatment...[that was] the most important.	Necessary. Without it we'd have to pull the plug.	Used for when kidneys aren't working. Cleans toxins...takes over for kidneys.	Toxins building up.	Tough one...Don't know.	[They] explained everything very well. Helped me make the right decision.

			LEGEND FOR COLUMN COLORS			
			denotes that participant mentioned toxin removal, cleaning, purification, or volume removal	denotes that participant mentioned accumalation of toxins or fluid as a reason for dialysis initiation	denotes responses that qualified as unaware or unable to identify alternatives (aside from death)	denotes a feeling of appreciation or gratitude towards renal physician or team
					denotes responses that qualified as aware of transplant or another modality	
					denotes responses that qualified as waiting longer for renal recovery	
